# Crystal structure, Hirshfeld surface analysis and spectroscopic characterization of the di-enol tautomeric form of the compound 3,3′-[(2-sulf­an­yl­idene-1,3-di­thiole-4,5-di­yl)bis­(sulfane­di­yl)]bis­(pentane-2,4-dione)

**DOI:** 10.1107/S2056989020010695

**Published:** 2020-08-11

**Authors:** Keysha T. Cordero Giménez, Victoria Y. Soto Díaz, Jean C. González Espiet, Alexis Lavín Flores, Jesbaniris Bas Concepción, Kevin E. Rivera Cruz, Dara L. Rodríguez Ayala, Dalice M. Piñero Cruz

**Affiliations:** a Department of Chemistry, University of Puerto Rico, Rio Piedras Campus, San Juan, 00927, Puerto Rico

**Keywords:** di­thio­les, redox state, non-innocent, tautomerism, crystal structure

## Abstract

The reaction between [TBA]_2_[Zn(dmit)_2_] and 3-chloro-2,4-penta­nedione yielded single crystals of the title compound, (3*E*,3′*E*)-3,3′-[(2-sulfanylidene-1,3-dithiole-4,5-diyl)bis(sulfanediyl)]bis(4-hydroxypent-3-en-2-one), C_13_H_14_O_4_S_5_, after solvent evaporation. The title compound crystallizes in the triclinic space group *P*  with two mol­ecules related by an inversion center present in the unit cell. The central thione ring moiety contains a carbon–carbon double bond covalently linked to two sulfoxide substituents located outside of the plane of the ring. The S—C—C—S torsion angles are −176.18 (8) and −0.54 (18)°. Intra­molecular hydrogen bonds occur within the two dione substituents (1.67–1.69 Å). Adjacent asymmetric units are linked by C—H⋯S (2.89–2.90 Å), S⋯S [3.569 (1) Å] and O⋯H [2.56–2.66 Å between non-stacked thione rings] short contacts.

## Abstract 2

A new di­thiol­ene derivative has been synthesized from [TBA]_2_[Zn(dmit)_2_] and 3-chloro-2,4-penta­nedione. Crystals were obtained by slow evaporation of an aceto­nitrile solution of the title compound, which crystallizes in the triclinic space group *P* 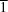. The structure of the S—C heterocycle includes two penta­dione moieties that are outside of the plane of the mol­ecule. Intra- and inter­molecular hydrogen bonds are observed, as well as C—H⋯S, S⋯S and O⋯H short contacts from other inter­molecular inter­actions.

## Chemical context   

Di­thiol­ene systems (McCleverty *et al.*, 1968 ▸) are a versatile family of compounds in coordination chemistry because of their redox non-innocent behavior (Eisenberg *et al.*, 2011 ▸). These compounds are electronically flexible and contribute to the stability of several redox processes observed in metal complexes, which are not necessarily ‘metal-based’ (Stiefel, 2004 ▸; Periyasamy *et al.*, 2007 ▸). Their electronic spin states can easily be clarified with the combined insights afforded by spectroscopic data, X-ray crystallography and computational analysis (Eisenberg *et al.*, 2011 ▸; Yan *et al.*, 2013 ▸; Lyaskovskyy *et al.*, 2012 ▸). Di­thiol­ene derivatives such as tetra­thia­fulvenyl-acetyl­acetonate (TTFSacacH), synthesized by Lorcy *et al.* (2001 ▸), have been reported as electroactive ligands with inter­esting redox properties. Most of these compounds employ the zinc–thiol­ate complex bis­(tetra­butyl­ammonium) bis­(1,3-di­thiole-2-thione-4,5-di­thiol­ato)zinc(II), [TBA]_2_[Zn(dmit)_2_]^2−^ (Comerlato *et al.* 2002 ▸), as a key starting material to achieve the synthesis of inter­esting metal complexes.
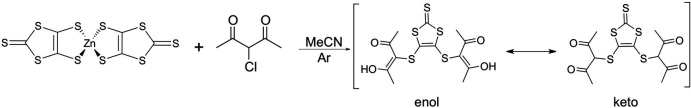


Herein, the reduction of the [Zn(dmit)_2_]^2−^ di­thiol­ene complex is utilized to aid the formation of a novel thio­carbonyl compound by its reaction with 3-chloro-2,4-penta­nedione (Cl-acac) to yield the title compound (3*E*,3′*E*)-3,3′-[(2-sulfanylidene-1,3-dithiole-4,5-diyl)bis(sulfanediyl)]bis(4-hydroxypent-3-en-2-one), the di-enol tautomer of 3,3′-[(2-sulf­an­yl­idene-1,3-di­thiole-4,5-di­yl)bis­(sulfane­di­yl)]bis­(pen­tane-2,4-dione). The electrophilic nature of the acetyl­acetone (acac) motif and the high electron density on the sulfur atoms drive the nucleophilic substitution to completion. The title compound is a double β-dicarbonyl compound that contains two acetyl­acetone moieties, which are found in their enolic form in the solid state. Concerning the reactivity of the title compound, it is able to undergo acid or base-catalyzed α-hydrogen substitution reactions, in which the rate-determining step is the formation of the enol or enolate anion (Shapet’ko *et al.*, 1975 ▸). Compared to the acid-catalyzed process, the self-enolization of most ketones is negligible. The double β-dicarbonyl compound described herein also undergoes tautomerization; however, in the solid phase, the enol tautomer predominates in this equilibrium as it is stabilized relative to the keto form *via* resonance through the conjugated π-system and by intra­molecular hydrogen bonding in the solid-state (Drexler *et al.* 1976 ▸; Seco *et al.* 1989 ▸). This aspect is confirmed by its FT–IR and NMR spectra.

## Structural commentary   

**Figure 1:**
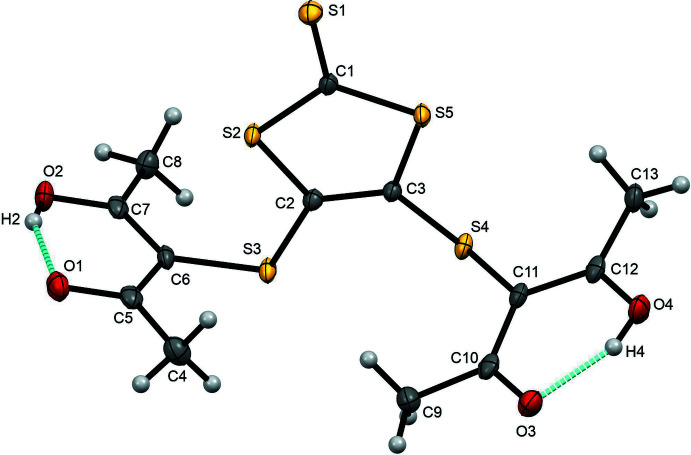
The title compound with displacement ellipsoids drawn at 50% probability level and hydrogen bonds (O—H⋯O) in the asymmetric unit indicated.

The title compound crystallizes in the triclinic space group *P* 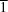 with one mol­ecule in the asymmetric unit (Fig. 1 ▸, Table 3 ▸). Its chemical structure consists of two 4-hy­droxy-3-penten-2-one units connected to a 1,3-di­thiol-1-thione ring moiety through a C—S single bond. In the unit cell, two mol­ecules are related by an inversion center. The central thione ring is conformed by a C2=C3 carbon–carbon double bond, which is in the same plane as the C1=S1 bond characteristic of the thione substituent. The angles C2—S3—C6 and C3—S4—C11 are 101.45 (7) and 103.72 (7)°, respectively. The torsion angles S4—C3—C2—S3 and S2—C2—C3—S5 are −176.18 (8) and −0.54 (18)°, respectively; the difference in the torsion angles is due to the effects of steric hindrance from the 4-hy­droxy-3-penten-2-one moiety. The S4—C3 and S3—C2 bond lengths are 1.7608 (16) and 1.7679 (16) Å, respectively.

## Supra­molecular features   

**Figure 2:**
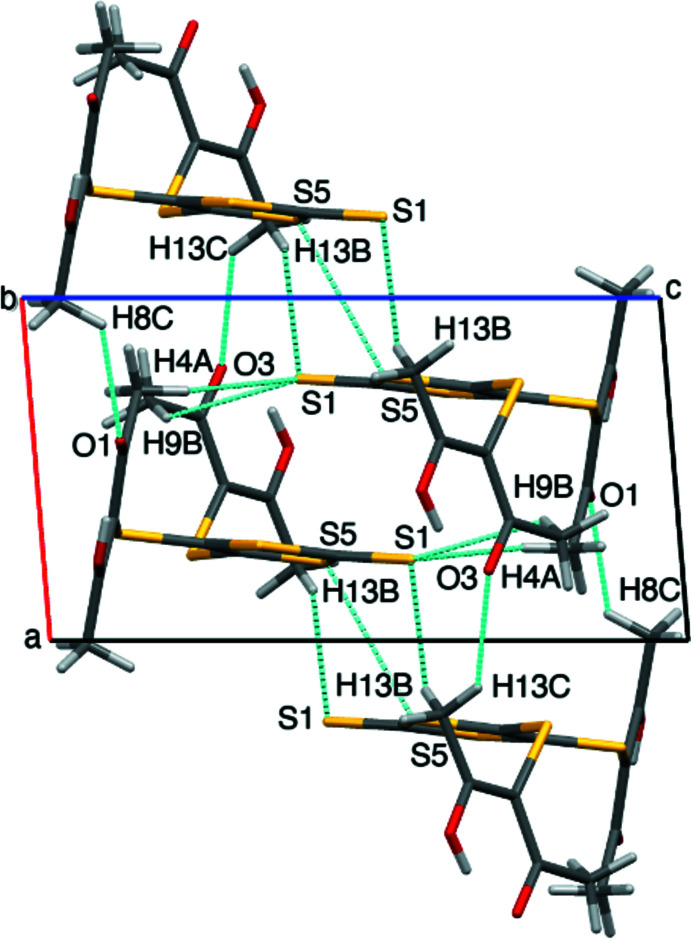
Crystal packing of the title compound, indicating the inter­molecular O⋯H—C, O⋯C and S⋯C, C—H⋯*S* and *S*⋯S short contacts, viewed along the *b* axis.

The title compound exhibits numerous inter­molecular inter­actions, namely four C—H⋯O, three C—H⋯S, three C⋯O, one S⋯C, and one S⋯S inter­action (Fig. 2 ▸, Tables 1 ▸ and 2 ▸). The five-membered thio­carbonyl-containing rings lie almost parallel to the *c* axis and extend in a sheet-like fashion, forming a network that propagates along the axis with all rings following the same orientation. The sheets are linked by out-of-plane C13—H13*B*⋯S1 short contacts, generating stacks along the *a* axis with S⋯S short contacts between adjacent mol­ecules [S5⋯S5^iv^ = 3.5688 (6) Å]. In addition, the nucleophilic atom S3 is oriented towards the electrophilic C5, leading to an S3⋯C5^iii^ [3.471 (2) Å] contact, further contributing to the extension of the network along the *c-*axis direction. Mol­ecules of the title compound also associate with neighboring mol­ecules above and below the thio­carbonyl ring planes through the acac backbone by C4—H4⋯S1 and C9—H9⋯S1 contacts. The acac backbone lies nearly perpendicular to the rings, and there are several key inter­actions between the carbonyl oxygen atoms (O1, O2, and O3) and neighboring methyl hydrogen atoms (H8*A* and H8*C*) with lengths in the range 2.56-2.66 Å. However, atom O4 is not involved in any inter­actions with hydrogen atoms, and instead makes short contacts with both C12 and C13.

## Hirshfeld Surface Analysis   

**Figure 3:**
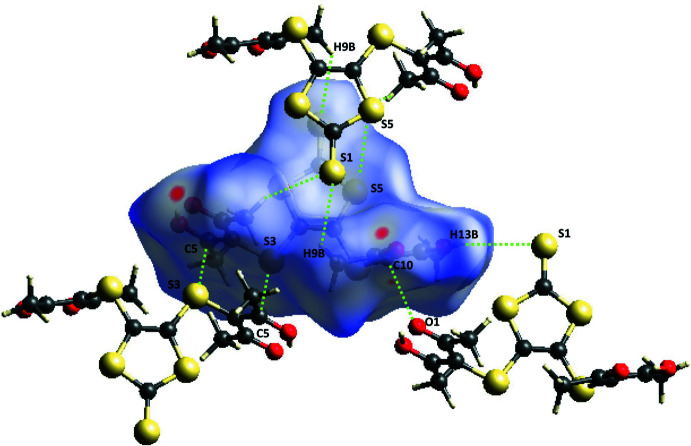
Hirshfeld surface of the title compound mapped over *d*_norm_ with the four main inter­molecular contacts in the crystal lattice shown.

**Figure 4:**
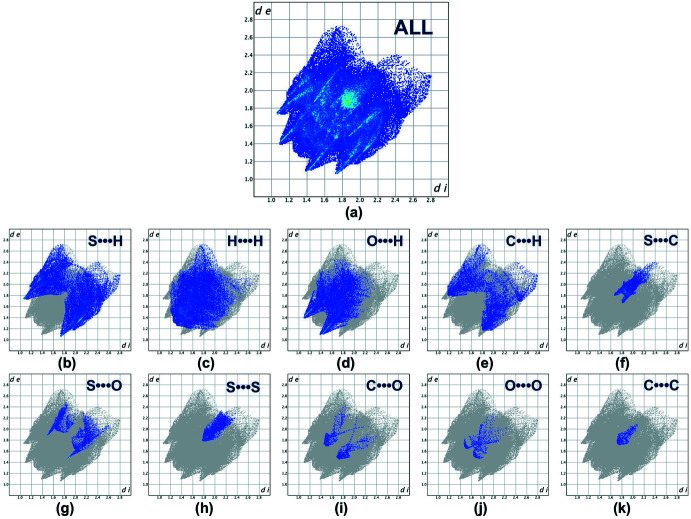
Full (*a*) and individual (*b*)–(*m*) two-dimensional fingerprint plots showing the ten inter­molecular contacts present in the crystal structure.

The Hirshfeld surface (Spackman & Jayatilaka, 2009 ▸) for the title compound mapped over *d*_norm_ is shown in Fig. 3 ▸ while Fig. 4 ▸ shows the associated two-dimensional fingerprint plots (McKinnon *et al.*, 2007 ▸), both generated with *CrystalExplorer17* (Turner *et al.*, 2017 ▸). Red spots on the Hirshfeld surface mapped over *d*_norm_ in the color range −0.0820 to 1.5568 arbitrary units confirm the above-mentioned primary inter­molecular contacts. The fingerprint plots are given for all contacts and those delineated into S⋯H/H⋯S (27.9%; Fig. 4 ▸ *b*), H⋯H (25.8%; Fig. 4 ▸ *c*), O⋯H/H⋯O (19.5%; Fig. 4 ▸ *d*), C⋯H/H⋯C (9.3%; Fig. 4 ▸ *e*), S⋯C/C⋯S (4.9%; Fig. 4 ▸ *f*), S⋯O/O⋯S (4.8%; Fig. 4 ▸ *g*), S⋯S (4.0%; Fig. 4 ▸ *h*), O⋯C/C⋯O (2.0%; Fig. 4 ▸ *i*), O⋯O (1.1%; Fig. 4 ▸ *j*), and C⋯C (0.7%; Fig. 4 ▸ *k*) inter­actions. Thus, the Hirshfeld surface analysis indicates that the most significant contributions arise from S⋯H and H⋯H contacts.

## Database survey   

A search of the Cambridge Structural Database (CSD Version 5.40, September 2019 update; Groom *et al.*, 2016 ▸) for the title compound revealed 46 hits comprising structures including metal complexes and organic compounds. Of the latter, 31 hits are for C–S bicyclic compounds and four hits are for monocyclic C–S crystal structures. Monocyclic structures related to the title compound are bis­(5-(mesityl­thio)-1,3-di­thiole-2-thione)-4,4′-di­sulfide dihydrate (LOBXEF; Cerrada *et al.*, 1999 ▸), 4,4′-disulfanediylbis{5-[(2,4,6-triiso­propyl­phen­yl)sulf­an­yl]-1,3-di­thiole-2-thione} (NUMXOJ; Cerrada *et al.*, 2009 ▸) and 4,5-bis­(2,4-di­nitro­phenyl­thio)-1,3-di­thiole-2-thione (YISBOR; Qi *et al.*, 1994 ▸ and YISBOR10; Qu *et al.*, 1995 ▸). The dihedral angles in YISBOR/YISBOR10 and LOBXEF are similar to those exhibited by the title compound, unlike in NUMXOJ, which is completely different. For the mentioned compounds, the lack of C—H⋯O and C—H⋯S short contacts means they are not comparable to the title compound. The four comparative compounds show similar S⋯C short contacts, which involve the sulfur atoms of the thione ring and the carbon atoms from the substituents. In contrast to YISBOR/YISBOR10, the structures of LOBXEF and NUMXOJ exhibit an S⋯S short contact analogous to that in the title compound. The structure of NUMXOJ exhibits π–π stacking of the thione rings of neighboring mol­ecules, similar to the title compound, and unlike LOBXEF (in which π-π stacking occurs between the benzene and thione rings) and YISBOR/YISBOR10 (where there π–π stacking between the thione ring and one benzene ring).

## Synthesis and crystallization   

The synthesis of the title compound was carried out by refluxing 1 eq. of [TBA]_2_[Zn(dmit)_2_] and 4 eq. of 3-chloro-2,4-penta­nedione in 100 mL of aceto­nitrile under argon for 1 h, after which it was cooled and left under stirring overnight. Activated carbon was added and stirring continued for 1 h. The resulting mixture was filtered and washed with cold aceto­nitrile. The solvent was evaporated under reduced pressure, and ethyl acetate was added to precipitate ZnCl_2_. The remaining solution was filtered, followed by evaporation of the solvent, giving a yellow crystalline solid (67.3% yield). NMR analyses were performed on a Bruker AV-700 spectrometer using chloro­form-*d* (CDCl_3_) as solvent. The solvent signals at 7.26 and 77.00 ppm were used as inter­nal standards for proton and carbon, respectively. ^1^H NMR (700 MHz, CDCl_3_) *δ* 15.40 (*s*, 1H, inter­changeable) 5.11(*s*, 1H, inter­changeable), 2.48 (*s*, 12H). ^13^C NMR (176 MHz, CDCl_3_) *δ* 24.92, 102.30, 131.40, 197.78, 209.18.

## Spectroscopic Characterization   

Without basic catalysis, the self-enolization of most ketones is negligible and the keto form is favored almost exclusively (Drexler *et al.*, 1976 ▸). However, β-dicarbonyl compounds, which can also undergo tautomerization, are stabilized in the enol tautomer *via* resonance of the conjugated π-system and intra­molecular hydrogen bonding. Furthermore, the enol is the less polar of the two tautomers because the intra­molecular hydrogen bond reduces the dipole–dipole repulsion of the two carbonyls in the keto form. The equilibrium of β-dicarbonyl compounds has been studied extensively and it has been shown that tautomeric inter­conversion between the diketo and enol forms is relatively slow and can be observed by NMR. Under normal conditions, the enolic form predominates in equilibrium (Egan *et al.*, 1977 ▸). This effect was demonstrated to be solvent and concentration dependent. An NMR study of keto–enol tautomerism in β-dicarbonyl compounds revealed that for the unsubstituted and symmetrical β-dicarbonyl compound pentane-2,4-dione, the equilibrium constant at 310 K has a value of 2.95 with 93.3 enol % (acetone exists as 0.00025% enol) (Schubert, 1960 ▸). In addition, as these compounds are progressively diluted with non-polar solvents, the enol content of the system increases. The progressive dilution with more polar solvents than the solute was observed to increase the stability of the keto form.

**Figure 5:**
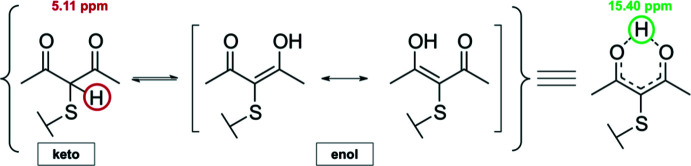
Tautomeric effect observed in the acetyl­acetonate portion of the title compound.

**Figure 6:**
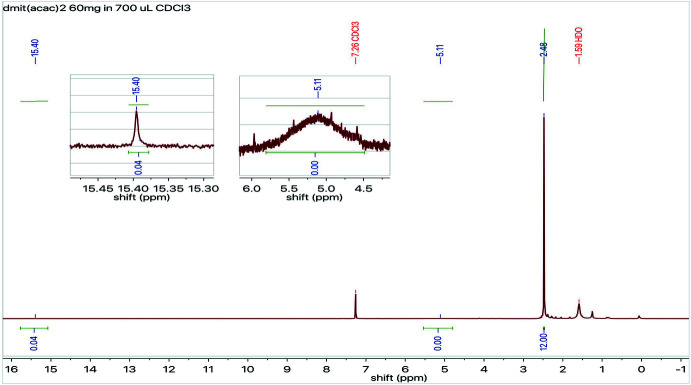
^1^H NMR spectrum of the title compound with inserts of the baseline expansions near 15.4 and 5.1 ppm, respectively.

In the case of the ^1^H NMR study of the title compound in deuterated chloro­form at 298 K, the predominant form was observed to be the enol tautomer. NMR was used to confirm the underlying symmetry the title compound possesses in solution, in which the enol tautomer predominates, as can be observed in Fig. 5 ▸. The lowest frequency signal in the ^1^H NMR spectrum integrates to twelve and corresponds to the methyl protons of the compound, indicating that the latter are chemically equivalent. Similarly, the enol form of the compound was observed crystallographically and in solution, exhibiting intra­molecular hydrogen bonding and renders both methyl groups, as well as both carbonyls, chemically equivalent. When studying the proton spectrum, the conjugation in the six-membered pseudo-aromatic ring deshields the signal of the inter­changeable proton, giving rise to a low field signal at 15.4 ppm that is lost in the baseline. Looking further into the baseline at higher fields, around 5.1 ppm, it reveals a wide signal that is almost lost in the noise and that can be assigned to the inter­changeable proton in the keto tautomer (Fig. 6 ▸). The formation of this hydrogen-bridge bond is promoted by the planar structure of the enol–carbonyl resonance system because this leads to an ideal spatial orientation of the hy­droxy group and carbonyl group in order to construct a strong hydrogen-bridge bond. Therefore, the monoenolic form of a β-dicarbonyl compound has a planar, six-membered cyclic structure stabilized by resonance. Decreasing the concentration of the solute in non-polar solvents has been proven to increase the concentration of the enol tautomer. ^13^C NMR spectrum displayed a single signal at 24.8 ppm for the methyl carbons, and a single signal at 197.7 ppm for the carbonyl carbons, supporting the statement that there is chemical equivalency between the methyl groups and, most importantly, between both carbonyl moieties. This effect has been previously demonstrated by comparing the ^13^C NMR spectra of the enol forms of symmetrical and unsymmetrical derivatives of β-diketones, where a different chemical shift was observed for the two carbonyls in the unsymmetrical case (Shapet’ko *et al.*, 1975 ▸). It is possible to conclude that the three signals of the 2,4-penta­nedione portion of the title compound, as well as the chemical shifts observed, are indicative of a symmetrical system that results from intra­molecular hydrogen-bonding in the enol tautomer.

**Figure 7:**
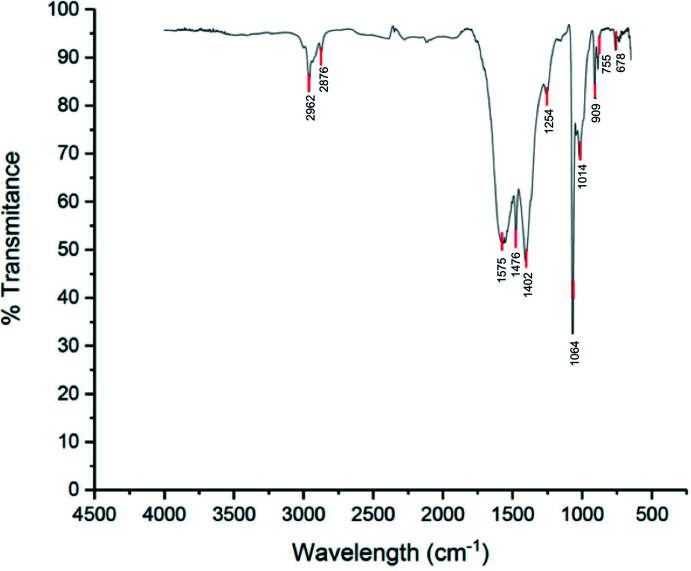
IR spectrum of the title compound.

IR peaks at 2,962 and 2,876 cm^−1^ are assigned to the C—H stretches (Fig. 7 ▸). The peaks between 1,575 and 1,402 cm^−1^ correspond to the C=C bond in the enol form. Moreover, hidden under this peak there is also the C=O stretch in the enol form, which is lowered by conjugation to the C=C bond and the O atom of the –OH group, respectively. OH stretches for β-diketones are tabulated from 3,200 to 2,400 cm^−1^; however, in the case of symmetric acac compounds where the enol form predominates and the inter­changeable hydrogen is located between the two carbonyls, the dipole change associated to the symmetric OH stretch is null, and the signal is minimal to non-existent. Thus, evidence from NMR and IR spectroscopy indicates that the compound exists almost entirely in its enol form.

## Refinement   

Crystal data, data collection and structure refinement details are summarized in Table 3 ▸. H atoms were included in geometrically calculated positions for the alkyl groups while the hydrogen atoms from OH groups were located from the difference-Fourier map and refined as riding: O—H = 0.82 Å, C—H = 0.93–0.98 Å with *U*_iso_(H) =1.5*U*_eq_(O, C-meth­yl) and 1.2*U*_eq_(C) for other H atoms.

## Supplementary Material

Crystal structure: contains datablock(s) I. DOI: 10.1107/S2056989020010695/dx2025sup1.cif

Structure factors: contains datablock(s) I. DOI: 10.1107/S2056989020010695/dx2025Isup2.hkl

CCDC reference: 1984599

Additional supporting information:  crystallographic information; 3D view; checkCIF report

## Figures and Tables

**Table 1:** C⋯O, S⋯C and S⋯S short contacts (Å)

C10⋯O1^i^	3.178 (2)	S3⋯C5^iii^	3.471 (2)
C12⋯O4^ii^	3.180 (2)	S5⋯S5^iv^	3.5688 (6)
C13⋯O4^ii^	3.219 (2)		

Symmetry codes: (i) 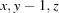; (ii) 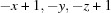; (iii) 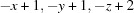; (iv) 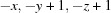.

**Table 2:** Hydrogen-bond geometry (Å, °)

*D*—H⋯*A*	*D*—H	H⋯*A*	*D*⋯*A*	*D*—H⋯*A*
O2—H2⋯O1	0.82	1.67	2.4228 (17)	151
O4—H4⋯O3	0.82	1.69	2.4406 (17)	151
C13—H13*C*⋯O3^v^	0.96	2.60	3.374 (3)	138
C8—H8*C*⋯O3^vi^	0.96	2.66	3.522 (2)	150
C8—H8*A*⋯O2^vii^	0.96	2.66	3.612 (2)	170
C8—H8*C*⋯O1^v^	0.96	2.56	3.228 (3)	127
C9—H9*B*⋯S1^viii^	0.96	2.90	3.5184 (19)	123
C13—H13*B*⋯S1^iv^	0.96	2.89	3.7165 (19)	144
C4—H4*A*⋯S1^viii^	0.96	2.90	3.7987 (19)	156

Symmetry codes: (iv) 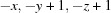; (v) 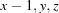; (vi) 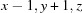; (vii) 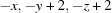; (viii) 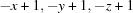.

**Table 3:** Experimental details

Crystal data	
Chemical formula	C_13_H_14_O_4_S_5_
*M*_r_	394.54
Crystal system, space group	Triclinic, *P* 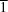
Temperature (K)	100
*a*, *b*, *c* (Å)	7.1843 (1), 9.9198 (1), 12.5230 (2)
α, β, γ (°)	84.319 (1), 83.574 (1), 69.151 (1)
*V* (Å^3^)	827.11 (2)
*Z*	2
Radiation type	Cu *K*α
μ (mm^−1^)	6.59
Crystal size (mm)	0.3 × 0.28 × 0.06

Data collection	
Diffractometer	Rigaku SuperNova HyPix3000
Absorption correction	Multi-scan (*CrysAlis PRO*; Rigaku OD, 2015 ▸)
*T*_min_, *T*_max_	0.208, 0.673
No. of measured, independent and observed [*I* > 2σ(*I*)] reflections	45972, 3086, 3034
*R*_int_	0.048
(sin θ/λ)_max_ (Å^−1^)	0.606

Refinement	
*R*[*F* ^2^ > 2σ(*F* ^2^)], *wR*(*F* ^2^), *S*	0.027, 0.073, 1.05
No. of reflections	3072
No. of parameters	206
H-atom treatment	H-atom parameters constrained
Δρ_max_, Δρ_min_ (e Å^−3^)	0.37, −0.31

Computer programs: *CrysAlis PRO* (Rigaku OD, 2015 ▸), *SHELXT2014/5* (Sheldrick, 2015*a* ▸), *SHELXL* (Sheldrick, 2015*b* ▸) and *OLEX2* (Dolomanov *et al.*, 2009 ▸).

## supplementary crystallographic information

### Crystal data

**Table:**

C_13_H_14_O_4_S_5_	*Z* = 2
*M**_r_* = 394.54	*F*(000) = 408
Triclinic, *P*1	*D*_x_ = 1.584 Mg m^−^^3^
*a* = 7.1843 (1) Å	Cu *K*α radiation, λ = 1.54184 Å
*b* = 9.9198 (1) Å	Cell parameters from 38605 reflections
*c* = 12.5230 (2) Å	θ = 3.6–68.9°
α = 84.319 (1)°	µ = 6.59 mm^−^^1^
β = 83.574 (1)°	*T* = 100 K
γ = 69.151 (1)°	Block, light yellow
*V* = 827.11 (2) Å^3^	0.3 × 0.28 × 0.06 mm

### Data collection

**Table:**

Rigaku SuperNova HyPix3000 diffractometer	3034 reflections with *I* > 2σ(*I*)
ω scans	*R*_int_ = 0.048
Absorption correction: multi-scan (CrysAlisPro; Rigaku OD, 2015)	θ_max_ = 69.1°, θ_min_ = 3.6°
*T*_min_ = 0.208, *T*_max_ = 0.673	*h* = −8→8
45972 measured reflections	*k* = −12→12
3086 independent reflections	*l* = −15→15

### Refinement

**Table:**

Refinement on *F*^2^	Secondary atom site location: difference Fourier map
Least-squares matrix: full	Hydrogen site location: mixed
*R*[*F*^2^ > 2σ(*F*^2^)] = 0.027	H-atom parameters constrained
*wR*(*F*^2^) = 0.073	*w* = 1/[σ^2^(*F*_o_^2^) + (0.0432*P*)^2^ + 0.5496*P*] where *P* = (*F*_o_^2^ + 2*F*_c_^2^)/3
*S* = 1.05	(Δ/σ)_max_ = 0.001
3072 reflections	Δρ_max_ = 0.37 e Å^−^^3^
206 parameters	Δρ_min_ = −0.31 e Å^−^^3^
0 restraints	Extinction correction: SHELXL-2016/6 (Sheldrick 2015b), Fc^*^=kFc[1+0.001xFc^2^λ^3^/sin(2θ)]^-1/4^
Primary atom site location: dual	Extinction coefficient: 0.0023 (3)

### Special details

**Table:**

Geometry. All esds (except the esd in the dihedral angle between two l.s. planes) are estimated using the full covariance matrix. The cell esds are taken into account individually in the estimation of esds in distances, angles and torsion angles; correlations between esds in cell parameters are only used when they are defined by crystal symmetry. An approximate (isotropic) treatment of cell esds is used for estimating esds involving l.s. planes.

### Fractional atomic coordinates and isotropic or equivalent isotropic displacement parameters (Å^2^)

**Table:**

	*x*	*y*	*z*	*U*_iso_*/*U*_eq_	
S1	0.23650 (6)	0.66587 (4)	0.42463 (3)	0.01782 (12)	
S2	0.27692 (6)	0.63562 (4)	0.65943 (3)	0.01461 (11)	
S3	0.31242 (6)	0.48313 (4)	0.88104 (3)	0.01525 (12)	
S4	0.24878 (6)	0.21427 (4)	0.76744 (3)	0.01430 (11)	
S5	0.22936 (6)	0.40181 (4)	0.55835 (3)	0.01485 (11)	
O1	0.58163 (18)	0.77059 (13)	0.86405 (10)	0.0219 (3)	
O2	0.22982 (18)	0.89341 (12)	0.91407 (10)	0.0208 (3)	
H2	0.348293	0.879765	0.896703	0.031*	
O3	0.79489 (17)	−0.06088 (12)	0.69638 (10)	0.0204 (3)	
O4	0.56236 (18)	−0.12438 (12)	0.60106 (10)	0.0212 (3)	
H4	0.667516	−0.126626	0.622140	0.032*	
C1	0.2480 (2)	0.57110 (16)	0.54050 (13)	0.0137 (3)	
C2	0.2827 (2)	0.48345 (16)	0.74276 (13)	0.0129 (3)	
C3	0.2616 (2)	0.37325 (16)	0.69608 (13)	0.0129 (3)	
C4	0.7301 (3)	0.5227 (2)	0.83018 (15)	0.0232 (4)	
H4A	0.728193	0.505999	0.756173	0.035*	
H4B	0.725804	0.439335	0.874947	0.035*	
H4C	0.850316	0.540141	0.839425	0.035*	
C5	0.5532 (3)	0.65102 (18)	0.86127 (13)	0.0175 (3)	
C6	0.3570 (2)	0.64631 (17)	0.88533 (13)	0.0146 (3)	
C7	0.1978 (2)	0.77374 (18)	0.91381 (13)	0.0163 (3)	
C8	−0.0110 (3)	0.78032 (18)	0.94305 (15)	0.0206 (4)	
H8A	−0.080210	0.861593	0.985597	0.031*	
H8B	−0.010564	0.692988	0.983764	0.031*	
H8C	−0.077272	0.790640	0.878697	0.031*	
C9	0.6980 (3)	0.13033 (18)	0.81362 (15)	0.0213 (4)	
H9A	0.632959	0.118292	0.883274	0.032*	
H9B	0.650861	0.230598	0.789056	0.032*	
H9C	0.839859	0.097756	0.818419	0.032*	
C10	0.6520 (2)	0.04366 (17)	0.73592 (14)	0.0170 (3)	
C11	0.4523 (2)	0.07528 (16)	0.70716 (13)	0.0148 (3)	
C12	0.4160 (2)	−0.01363 (17)	0.63824 (14)	0.0168 (3)	
C13	0.2157 (3)	0.00525 (18)	0.60412 (15)	0.0203 (4)	
H13A	0.231343	−0.053312	0.544829	0.030*	
H13B	0.148642	0.104954	0.582310	0.030*	
H13C	0.137979	−0.023707	0.663218	0.030*	

### Atomic displacement parameters (Å^2^)

**Table:**

	*U*^11^	*U*^22^	*U*^33^	*U*^12^	*U*^13^	*U*^23^
S1	0.0235 (2)	0.0160 (2)	0.0145 (2)	−0.00766 (16)	−0.00267 (16)	0.00069 (15)
S2	0.0234 (2)	0.00936 (19)	0.0133 (2)	−0.00776 (15)	−0.00300 (15)	−0.00126 (14)
S3	0.0252 (2)	0.0117 (2)	0.0117 (2)	−0.00935 (15)	−0.00231 (15)	−0.00187 (14)
S4	0.0192 (2)	0.00921 (19)	0.0154 (2)	−0.00658 (15)	0.00134 (15)	−0.00193 (15)
S5	0.0223 (2)	0.01119 (19)	0.0137 (2)	−0.00800 (15)	−0.00379 (15)	−0.00224 (14)
O1	0.0268 (6)	0.0210 (6)	0.0239 (7)	−0.0153 (5)	−0.0035 (5)	−0.0008 (5)
O2	0.0266 (6)	0.0135 (6)	0.0254 (7)	−0.0100 (5)	−0.0020 (5)	−0.0051 (5)
O3	0.0195 (6)	0.0155 (6)	0.0250 (7)	−0.0051 (5)	−0.0005 (5)	−0.0012 (5)
O4	0.0227 (6)	0.0145 (6)	0.0259 (7)	−0.0046 (5)	−0.0007 (5)	−0.0076 (5)
C1	0.0137 (7)	0.0102 (7)	0.0173 (8)	−0.0036 (6)	−0.0020 (6)	−0.0029 (6)
C2	0.0153 (7)	0.0113 (7)	0.0120 (8)	−0.0046 (6)	−0.0007 (6)	−0.0013 (6)
C3	0.0161 (7)	0.0101 (7)	0.0127 (8)	−0.0047 (6)	−0.0016 (6)	−0.0011 (6)
C4	0.0216 (8)	0.0263 (9)	0.0233 (9)	−0.0089 (7)	−0.0015 (7)	−0.0072 (7)
C5	0.0247 (8)	0.0186 (8)	0.0114 (8)	−0.0096 (7)	−0.0048 (6)	−0.0006 (6)
C6	0.0223 (8)	0.0128 (7)	0.0119 (8)	−0.0093 (6)	−0.0030 (6)	−0.0022 (6)
C7	0.0256 (8)	0.0161 (8)	0.0097 (8)	−0.0093 (7)	−0.0047 (6)	−0.0011 (6)
C8	0.0224 (8)	0.0163 (8)	0.0227 (9)	−0.0061 (7)	−0.0001 (7)	−0.0041 (7)
C9	0.0256 (9)	0.0185 (8)	0.0221 (9)	−0.0089 (7)	−0.0070 (7)	−0.0017 (7)
C10	0.0237 (8)	0.0110 (7)	0.0166 (8)	−0.0076 (6)	−0.0004 (6)	0.0027 (6)
C11	0.0194 (8)	0.0083 (7)	0.0166 (8)	−0.0055 (6)	0.0004 (6)	−0.0008 (6)
C12	0.0236 (8)	0.0105 (7)	0.0170 (8)	−0.0079 (6)	0.0013 (6)	0.0004 (6)
C13	0.0246 (8)	0.0137 (8)	0.0252 (9)	−0.0091 (7)	−0.0021 (7)	−0.0047 (7)

### Geometric parameters (Å, º)

**Table:**

S1—C1	1.6424 (17)	C4—H4C	0.9600
S2—C1	1.7389 (16)	C4—C5	1.491 (2)
S2—C2	1.7385 (16)	C5—C6	1.424 (2)
S3—C2	1.7679 (16)	C6—C7	1.415 (2)
S3—C6	1.7653 (16)	C7—C8	1.483 (2)
S4—C3	1.7608 (16)	C8—H8A	0.9600
S4—C11	1.7700 (16)	C8—H8B	0.9600
S5—C1	1.7225 (16)	C8—H8C	0.9600
S5—C3	1.7479 (16)	C9—H9A	0.9600
O1—C5	1.278 (2)	C9—H9B	0.9600
O2—H2	0.8200	C9—H9C	0.9600
O2—C7	1.287 (2)	C9—C10	1.495 (2)
O3—C10	1.267 (2)	C10—C11	1.435 (2)
O4—H4	0.8200	C11—C12	1.399 (2)
O4—C12	1.303 (2)	C12—C13	1.489 (2)
C2—C3	1.351 (2)	C13—H13A	0.9600
C4—H4A	0.9600	C13—H13B	0.9600
C4—H4B	0.9600	C13—H13C	0.9600
			
C10···O1^i^	3.178 (2)	S3···C5^iii^	3.471 (2)
C12···O4^ii^	3.180 (2)	S5···S5^iv^	3.5688 (6)
C13···O4^ii^	3.219 (2)		
			
C2—S2—C1	97.02 (7)	C6—C7—C8	123.84 (15)
C6—S3—C2	101.45 (7)	C7—C8—H8A	109.5
C3—S4—C11	103.72 (7)	C7—C8—H8B	109.5
C1—S5—C3	97.48 (7)	C7—C8—H8C	109.5
C7—O2—H2	109.5	H8A—C8—H8B	109.5
C12—O4—H4	109.5	H8A—C8—H8C	109.5
S1—C1—S2	122.31 (9)	H8B—C8—H8C	109.5
S1—C1—S5	124.58 (10)	H9A—C9—H9B	109.5
S5—C1—S2	113.10 (9)	H9A—C9—H9C	109.5
S2—C2—S3	118.79 (9)	H9B—C9—H9C	109.5
C3—C2—S2	116.65 (13)	C10—C9—H9A	109.5
C3—C2—S3	124.56 (13)	C10—C9—H9B	109.5
S5—C3—S4	120.39 (9)	C10—C9—H9C	109.5
C2—C3—S4	123.78 (13)	O3—C10—C9	118.05 (15)
C2—C3—S5	115.69 (12)	O3—C10—C11	120.25 (15)
H4A—C4—H4B	109.5	C11—C10—C9	121.69 (14)
H4A—C4—H4C	109.5	C10—C11—S4	120.87 (12)
H4B—C4—H4C	109.5	C12—C11—S4	119.52 (12)
C5—C4—H4A	109.5	C12—C11—C10	119.34 (14)
C5—C4—H4B	109.5	O4—C12—C11	120.13 (15)
C5—C4—H4C	109.5	O4—C12—C13	115.14 (14)
O1—C5—C4	117.29 (15)	C11—C12—C13	124.71 (15)
O1—C5—C6	119.46 (15)	C12—C13—H13A	109.5
C6—C5—C4	123.24 (15)	C12—C13—H13B	109.5
C5—C6—S3	120.55 (12)	C12—C13—H13C	109.5
C7—C6—S3	120.13 (12)	H13A—C13—H13B	109.5
C7—C6—C5	119.32 (14)	H13A—C13—H13C	109.5
O2—C7—C6	119.97 (15)	H13B—C13—H13C	109.5
O2—C7—C8	116.18 (15)		
			
S2—C2—C3—S4	−176.18 (8)	C2—S3—C6—C5	−84.62 (14)
S2—C2—C3—S5	−0.54 (18)	C2—S3—C6—C7	95.98 (14)
S3—C2—C3—S4	3.1 (2)	C3—S4—C11—C10	80.09 (14)
S3—C2—C3—S5	178.72 (8)	C3—S4—C11—C12	−105.92 (14)
S3—C6—C7—O2	−178.14 (12)	C3—S5—C1—S1	178.43 (11)
S3—C6—C7—C8	1.0 (2)	C3—S5—C1—S2	−2.54 (10)
S4—C11—C12—O4	−173.78 (12)	C4—C5—C6—S3	0.8 (2)
S4—C11—C12—C13	4.5 (2)	C4—C5—C6—C7	−179.82 (15)
O1—C5—C6—S3	179.45 (12)	C5—C6—C7—O2	2.4 (2)
O1—C5—C6—C7	−1.1 (2)	C5—C6—C7—C8	−178.41 (15)
O3—C10—C11—S4	176.35 (12)	C6—S3—C2—S2	−6.57 (11)
O3—C10—C11—C12	2.3 (2)	C6—S3—C2—C3	174.19 (14)
C1—S2—C2—S3	179.61 (9)	C9—C10—C11—S4	−2.8 (2)
C1—S2—C2—C3	−1.09 (14)	C9—C10—C11—C12	−176.84 (15)
C1—S5—C3—S4	177.69 (10)	C10—C11—C12—O4	0.3 (2)
C1—S5—C3—C2	1.89 (14)	C10—C11—C12—C13	178.60 (15)
C2—S2—C1—S1	−178.63 (10)	C11—S4—C3—S5	63.50 (11)
C2—S2—C1—S5	2.31 (10)	C11—S4—C3—C2	−121.05 (15)

Symmetry codes: (i) *x*, *y*−1, *z*; (ii) −*x*+1, −*y*, −*z*+1; (iii) −*x*+1, −*y*+1, −*z*+2; (iv) −*x*, −*y*+1, −*z*+1.

### Hydrogen-bond geometry (Å, º)

**Table:**

*D*—H···*A*	*D*—H	H···*A*	*D*···*A*	*D*—H···*A*
O2—H2···O1	0.82	1.67	2.4228 (17)	151
O4—H4···O3	0.82	1.69	2.4406 (17)	151
C13—H13*C*···O3^v^	0.96	2.60	3.374 (3)	138
C8—H8*C*···O3^vi^	0.96	2.66	3.522 (2)	150
C8—H8*A*···O2^vii^	0.96	2.66	3.612 (2)	170
C8—H8*C*···O1^v^	0.96	2.56	3.228 (3)	127
C9—H9*B*···S1^viii^	0.96	2.90	3.5184 (19)	123
C13—H13*B*···S1^iv^	0.96	2.89	3.7165 (19)	144
C4—H4*A*···S1^viii^	0.96	2.90	3.7987 (19)	156

Symmetry codes: (iv) −*x*, −*y*+1, −*z*+1; (v) *x*−1, *y*, *z*; (vi) *x*−1, *y*+1, *z*; (vii) −*x*, −*y*+2, −*z*+2; (viii) −*x*+1, −*y*+1, −*z*+1.

### Selected bond lengths (Å) and angles (°)

**Table:**

C1=S1	1.6424 (17)
C2=C3	1.351 (2)
S2-C2	1.7385 (16)
S3-C2	1.7679 (16)
S5-C3	1.7479 (16)
S4-C3	1.7608 (16)
O1=C5	1.278 (2)
O2-C7	1.287 (2)
O3=C10	1.267 (2)
O4-C12	1.303 (2)
O2-H2	0.8200
O4-H4	0.8200
S2-C2-S3	118.79 (9)
S5-C3-S4	120.39 (9)
C6-S3-C2	101.45 (7)
C3-S4-C11	103.72 (7)
C7-C6-C5	119.32 (14)
C12-C11-C10	119.34 (14)
